# Perceived severity of forward versus backward balance disturbances during walking in young and older adults

**DOI:** 10.1371/journal.pone.0317943

**Published:** 2025-11-20

**Authors:** Shreya Jain, Nicolas Schweighofer, James M. Finley

**Affiliations:** 1 Division of Biokinesiology and Physical Therapy, University of Southern California, Los Angeles, California, United States of America; 2 Neuroscience Graduate Program, University of Southern California, Los Angeles, California, United States of America; 3 Department of Biomedical Engineering, University of Southern California, Los Angeles, California, United States of America; Pennsylvania State University Main Campus: The Pennsylvania State University - University Park Campus, UNITED STATES OF AMERICA

## Abstract

Falls, which often result from trips or slips, pose a major health concern, particularly among older adults. Experiencing falls or near-falls from balance disturbances can shape subsequent gait-related decisions, as individuals may avoid situations they perceive as risky or dangerous. Here, we explore whether perceptions of the severity of a gait disturbance are sensitive to the direction of the resulting loss of balance – forward or backward – and whether these perceptions change with age. Twenty young and twenty older adults walked on a split-belt treadmill while performing a two-alternative forced-choice task, where they indicated their preference between a forward-falling and a backward-falling treadmill perturbation. We varied the perturbation magnitudes using an adaptive staircase algorithm to obtain multiple forward-backward equivalence points, which reflect the points at which a forward and a backward perturbation are perceived as being equally severe. Using a mixed-effects linear model, we estimated the slope of this relationship between forward and backward treadmill perturbations, which quantified the direction and strength of the sensitivity to perturbation type. To assess reliability, we repeated the procedure on a second day. Additionally, we investigated two potential reasons underlying any observed sensitivity – 1) emotional responses measured by state anxiety, and 2) physical responses measured by peak center of mass velocity. We found that both young and older adults perceived backward-falling perturbations to be more severe than forward-falling ones, with no group difference in sensitivity. This sensitivity was moderately reliable across two days of testing, though most participants were less sensitive to perturbation direction on the second day. Neither state anxiety responses nor peak center of mass (CoM) velocity explained the directional sensitivity, though deviations in peak CoM velocity from unperturbed walking were higher during backward-falling than forward-falling perturbations for both age groups. These results suggest that the perceived severity of a gait disturbance is not determined solely by its magnitude, but also by its direction, thereby making direction an important component of gait-related decisions. Integrating a measure of relative perception of types of gait disturbances with the ability to recover from them may provide a more comprehensive assessment of fall risk and inform personalized training interventions tailored to individual perceptions and preferences.

## Introduction

Falls are a public health concern, particularly among older adults for whom they can lead to devastating injuries and significant reductions in quality of life [1–3]. Falls may result from several risk factors, including intrinsic factors such as physical and cognitive ability, extrinsic factors such as features of the environment, and behavioral factors such as risk-taking [4–6]. The decision to engage in potentially risky behaviors, such as walking in crowded or cluttered areas, relies on one’s perception of the level of risk in the current environment. Risk perception is a subjective assessment of the possible outcomes that may occur in a given situation, in terms of their severity and probability [7,8]. In the context of walking, these outcomes may be losses of balance due, for example, to tripping over an obstacle or slipping, which are common causes of falls in both young and older adults [2,9–11]. While studies have extensively examined the recovery mechanisms involved in responding to forward and backward balance perturbations [12–25], how these are perceived relative to each other and how this perceived risk informs future behavior remains poorly understood.

Recovery from forward versus backward perturbations involves distinct biomechanical strategies. Recovery from forward-falling trips typically involves rapidly swinging the perturbed limb forward or elevating it over the obstacle to reposition the base of support ahead of the displaced center of mass (CoM), using momentum to aid in balance recovery [18,23,26,27]. In contrast, backward-falling slips often require braking or retracting the slipping foot to prevent further displacement [28–30], stepping with the non-slipping foot [31,32], and stabilizing the upper body to counter backward CoM movement [28,30,32]. Several studies suggest that slips pose greater recovery challenges than trips due to the rapid and often unexpected loss of friction, which leads to larger CoM displacements and higher corrective demands on the body [33,34]. Given these biomechanical differences, it is possible that backward disturbances are perceived as more severe or riskier than forward disturbances, as the greater difficulty in recovering from the former may inform individuals’ subjective assessments of their potential consequences.

Forward and backward balance perturbations have been studied by simulating them using a split-belt treadmill [35–41]. Forward perturbations are most commonly delivered by either rapidly stopping one belt [35] or by rapidly accelerating one belt [37], both of which have been shown to induce a similar effect on the CoM when compared to overground trips such that there is an increase in forward CoM velocity, angular momentum, and an anterior shift in the CoM relative to the base of support [35,37,41]. Backward perturbations are most commonly delivered by decelerating a treadmill belt to a slower speed or reversing the treadmill belt direction, causing a displacement of the CoM in the backward direction [35,41]. While such responses to these perturbations have been investigated, mechanisms used to perceive them are not as well understood. A recent study identifying perceptual thresholds for backward perturbations on the treadmill found that whole-body CoM velocity is an important source of sensory information that may be used to perceive these gait disturbances [39]. Therefore, it is possible that this may also be an important signal for perception of forward perturbations, and may inform perceptions of severity of different types of perturbations relative to each other.

Risk perception is inherently a subjective process and is strongly influenced by emotional responses to risky situations [8,42]. Therefore, risk perception in the context of walking with balance disturbances may also be influenced by specific emotions evoked by these perturbations. Studies of static balance have found that postural threat (e.g., standing at the edge of an elevated platform) leads to elevated state anxiety, which is a temporary emotional response to a given situation, and this anxiety response scales with the level of threat [43–46]. When standing in a higher threat condition, individuals perceive their forward lean to be greater than its true value, thereby perceiving their postural risk to be higher than it is [45]. While the influence of state anxiety on perception of risk during gait have not directly been studied, it has been found to be associated with more cautious locomotor behavior such as reduced walking speed and increased body rotation during obstacle negotiation [47,48]. When walking with balance disturbances, the relationship between any induced state anxiety responses, recovery responses and the perception of these disturbances remains to be understood. If state anxiety during disturbed gait scales with the level of perceived risk, and forward and backward perturbations are perceived differently in terms of their severity, these emotional responses might explain this difference between perturbation types.

Older adults are often more risk-averse than young adults in several domains of decision-making [49–51]. In situations concerning health, safety, and recreational activities, older adults report perceiving more risk than young adults [52], but whether these trends extend to gait and balance related decisions remains to be seen. Epidemiological studies of falls in older adults often combine falls related to forward-falling trips and backward-falling slips into one category [5,11], even though they occur by distinct mechanisms. Studies that have separated these have found mixed results, with one reporting a higher incidence of slip-induced falls and another reporting a higher incidence of trip-induced falls [53,54]. In the latter study, however, older adults reported attempting to take a protective step in 82% of forward falls while this was attempted in only 37% of backward falls [53]. These findings suggest that although trips may occur more frequently, slips may pose a greater threat due to the lower likelihood of successful recovery, particularly in older adults. Age-related declines in reactive stepping responses [14,33,55] and lower limb strength [56–58] further reduce the ability of older adults to recover from backward-falling slips, potentially heightening the perceived risk associated with backward perturbations.

The objective of this study was to assess the relative perceived risk of forward versus backward balance disturbances during walking in young and older adults, using perturbations on a split-belt treadmill. We hypothesized that backward-falling treadmill perturbations would be perceived to be riskier than forward-falling treadmill perturbations, and this sensitivity to perturbation direction would be greater in older adults than young adults. A secondary objective of this study was to investigate if changes in whole-body CoM kinematics or measures of state anxiety explained the observed differences in perceived risk between the two directions of perturbations. We hypothesized that differences in CoM responses between forward and backward perturbations would correlate with any observed sensitivity in perceived risk. Lastly, since state anxiety influences risk perception and postural control in threatening conditions, we hypothesized that differences in self-reported state anxiety in response to forward and backward perturbations would correlate with relative perceived risk between the two perturbation types. Understanding how these two common perturbation types are perceived relative to each other may ultimately help identify targets for fall prevention and perturbation training interventions that are better aligned with people’s perceptions of their own experiences.

## Methods

### Experimental procedures

Twenty young adults (11 female, 27 ± 5 years) and twenty older adults (12 female, 71 ± 4 years) participated in this study. Participants were included if they were between 18–35 years of age (young adults) or 65–85 years of age (older adults), had no current injuries or pain affecting walking ability, no diagnosed neurological conditions, and no uncontrolled hypertension. No formal sample size calculation was performed, but a target of 20 participants per group was selected based on feasibility considerations and consistency with sample sizes in similar locomotor perturbation studies [38,39]. All participants provided their written informed consent in accordance with a protocol approved by the Institutional Review Board of the University of Southern California (Protocol UP-22–00317).

### Clinical and self-report assessments

Participants completed two visits spaced at least a week apart, an interval chosen to minimize immediate carryover effects between sessions while allowing sufficient time for recovery. We began the first visit by having them complete a set of clinical assessments. We assessed overall balance ability using the MiniBESTest [59]. The assessment consists of 14 items with a maximum possible score of 28, divided into four subscales that assess anticipatory postural control, reactive postural control, sensory orientation, and stability during gait. This was followed by the Activities-specific Balance Confidence (ABC) scale [60] as a measure of perceived balance ability, and a History of Falls questionnaire [11] to record falls that occurred in the 12 months preceding the visit. To estimate physical activity levels, participants provided self-reports of the approximate time in minutes per week that they engage in exercise, using a single-question self-assessment.

### Self-selected walking speed

We determined participants’ self-selected walking speed for both overground and treadmill walking. Overground walking speed was determined using an average of three 10-meter walks timed using a stopwatch. To determine self-selected walking speed on the treadmill (Fully Instrumented Treadmill, Bertec Corporation, Columbus, OH, USA), we used an adaptive staircase algorithm [61]. Starting at 80% of the overground speed, treadmill speed was first increased by steps of 0.1 m/s for 10 s until the participant verbally indicated that they were at their comfortable walking speed. Next, the speed was further increased by 0.1 m/s, followed by a down-ramp with a step size of 0.1 m/s until the participant verbally indicated that they were at their preferred walking speed. The up-ramp and down-ramp were repeated a second time with a step size of 0.05 m/s. The final self-selected walking speed was computed as the average of the four indicated preferred walking speeds. This procedure was followed by an acclimation trial with two minutes of walking at the determined self-selected walking speed.

### Familiarization with perturbations

To determine differences in perceived risk of forward versus backward balance disturbances, we accelerated or decelerated the belts of a split-belt treadmill while participants walked [35,38,39,61,62]. Perturbations were delivered by rapidly changing the speed of one belt during the swing phase, which was detected in real time based on the vertical ground reaction force dropping below 80N [61]. Once detected, the target belt was accelerated or decelerated at 3m/s^2^ until it reached the specified perturbation speed. The new speed was held constant for 700ms, ensuring the belt speed remained changed throughout the step, after which it returned to the self-selected walking speed. Faster belt speeds led to forward-falling perturbations, and slower belt speeds led to backward-falling perturbations. All participants wore a safety harness throughout the experiment. While handlebars were attached at the front of the treadmill, participants were verbally discouraged from using them for external support. Despite this, a small number of individuals occasionally made brief contact, but these trials were not excluded. Additionally, there were no instances of falls into the safety harness, indicating that perturbation magnitudes were challenging but safe.

On the first day of testing, participants underwent a short familiarization procedure to attenuate any effects of the novelty of split-belt treadmill perturbations on choices. This included two familiarization blocks, one with forward perturbations and one with backward perturbations, with the order of these blocks counterbalanced between participants. One familiarization block consisted of two minutes of walking with two perturbations, each with a magnitude of 0.6 m/s, with this magnitude reflecting the change in treadmill belt speed from self-selected walking speed. This magnitude was chosen based on pilot observations as a clearly perceptible yet safe perturbation that would help prepare participants for the range of perturbations used in the main experiment. After each block, participants responded to a State Anxiety Scale, which was used to assess their emotional response to both directions of perturbations [63]. This allowed us to test if differences in the anxiety response could explain any observed differences in the perceived risk of the two perturbation directions. The State Anxiety Scale consisted of 9 items related to somatic anxiety (5 items: ‘*I felt nervous’; ‘My body was tense’; ‘I felt my stomach sinking’; ‘My heart was racing’; ‘I found myself hyperventilating’*), concentration disruption (2 items: ‘*I had lapses of concentration’; ‘Thoughts of falling interfered with my concentration’*), and worry (2 items: ‘*I had self-doubts’; ‘I was worried about my personal safety’*), which were scored on a 9-point Likert scale, ranging from 1 (*‘I do not feel this at all’*) to 9 (*‘I feel this extremely’*).

### Two-alternative forced choice task

**Fig 1:**
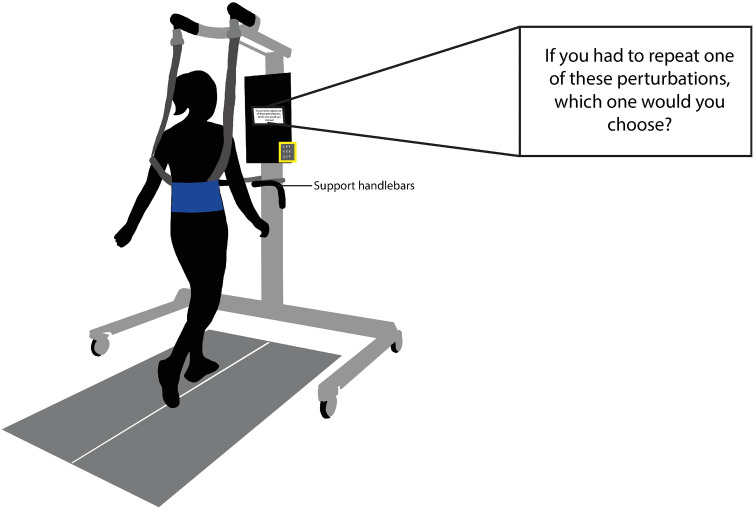
Experimental setup. Participants walked on a split-belt treadmill and received forward-falling and backward-falling perturbations. The decision prompt (enlarged on the top right) appeared on a monitor in front of the treadmill, and participants indicated their choices using a keypad (highlighted in the yellow box).

To quantify differences in perceived risk based on perturbation direction, we used a two-alternative forced-choice task wherein participants experienced one forward and one backward perturbation and then responded to the prompt - “If you had to repeat one of these perturbations, which one would you choose?” The prompt appeared on a monitor placed at the front of the treadmill, and participants indicated their choice using a keypad (Fig 1). Because there was no incentive provided for choosing the worse outcome, we assumed that this question prompted participants to choose the perturbation that was perceived to be less risky. We verified this assumption by asking participants to verbalize their decision-making process at the end of the study.

**Fig 2:**
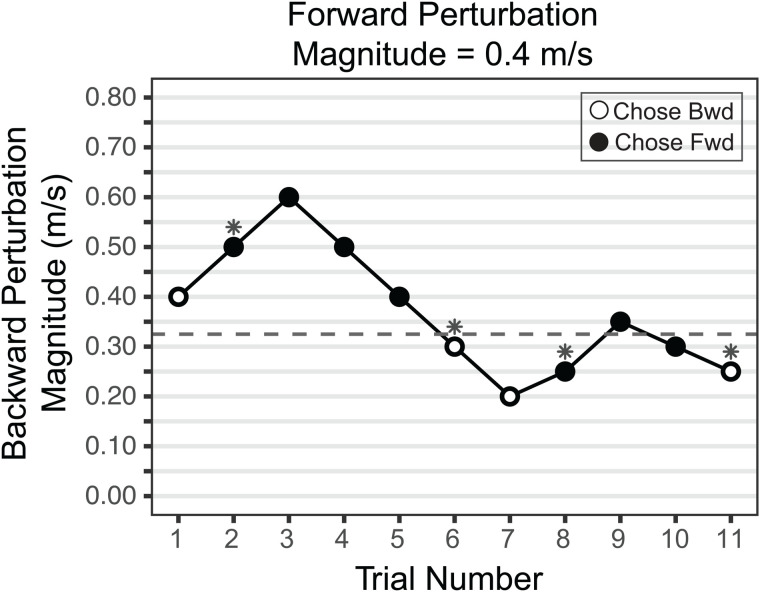
Example of the staircase procedure for a forward perturbation (Fwd) magnitude of 0.4m/s. Each point refers to one choice between a forward perturbation of 0.4 m/s magnitude, and a backward perturbation (Bwd) of the magnitude indicated on the y-axis. Open circles indicate a choice of the backward perturbation, and filled circles indicate a choice of the forward perturbation. At trial 1, both perturbations were of the same magnitude. For each up-ramp, the inflection point is the trial at which they chose the forward perturbation (filled circle). For each down-ramp, it is when they chose the backward perturbation (open circle). Inflection points, indicated by asterisks, occurred during trials 2, 6, 8, and 11, and the equivalence point was computed as the average of the four inflection points. In this example, the equivalence point is 0.325 (dashed horizontal line), implying that a backward perturbation of 0.325m/s is perceived to be equivalent to a forward perturbation of 0.4m/s.

We used seven different forward perturbation magnitudes ranging from 0.3 to 0.6 m/s in increments of 0.05 m/s. This range was selected based on pilot testing, where participants reported these magnitudes as perceptible yet tolerable, without any adverse events. In addition, constraining the forward perturbation range served to limit the backward perturbations produced by the staircase procedure, further helping to ensure participant safety. For each forward perturbation magnitude, an adaptive staircase procedure was used to determine the equivalent preferred backward perturbation magnitude, referred to here as the equivalence point [64,65]. The staircase started with a forward and a backward perturbation of equal magnitude and consisted of two up-ramps and two down-ramps. In the first up-ramp, the backward perturbation magnitude was increased in steps of 0.1 m/s until the participant chose the forward perturbation, marking the first inflection point. The backward perturbation magnitude was then increased by an additional 0.1 m/s to start the first down-ramp. During this down-ramp, the backward perturbation magnitude was decreased in 0.1 m/s steps until the participant chose the backward perturbation, marking the second inflection point. The second up-ramp then began starting at 0.1 m/s below the second inflection point, and the second down-ramp began at 0.1 m/s above the third inflection point. In these latter two ramps, the step size was reduced to 0.05 m/s to refine the estimate. An example of the staircase procedure for a forward perturbation magnitude of 0.4 m/s is shown in Fig 2. The equivalence point was computed as the average of the four inflection points. The number of trials or forward-backward perturbation pairs within each staircase was dependent upon participants’ choices and therefore, varied within and between participants. The time interval between two perturbations within a trial was 2s, after which the next swing phase of the target foot was detected to trigger the second perturbation. The time interval between two trials was 5s.

This staircase procedure was repeated on both the left and right sides, yielding 14 equivalence points between treadmill trips and slips for each participant. To assess the reliability of this procedure and the stability of the equivalence points, we repeated this procedure on a second visit at least a week after the first one. At the end of the second visit, participants provided a subjective description of their decision-making strategy.

### Data acquisition

While participants walked, positions of the pelvis and feet were recorded with an 11-camera Qualisys Oqus camera system (Qualisys AB, Göteborg, Sweden), using a configuration of thirteen reflective markers – one on each greater trochanter, three on the pelvis (posterior superior iliac spines, lumbosacral joint) and four on each foot (fifth metatarsal, lateral malleolus, first toe, heel). A static calibration trial was performed at the beginning of each session with additional markers placed on bilaterial anterior superior iliac spines, iliac crests, medial malleoli and first metatarsals to define the pelvis and foot segments. These markers were removed before the dynamic trials began. Due to marker drop-off, these data were available and analyzed for 18 young and 19 older adults.

### Data processing

We post-processed the kinematic data in Visual3D (C-Motion, Rockville, MD, USA) and MATLAB R2022b (MathWorks, USA). Marker positions were low-pass filtered using a 4th order Butterworth filter with a cutoff frequency of 6 Hz [66–68]. Heel strike and toe off events were identified as the peak anterior position of the heel marker and peak posterior position of the toe marker, respectively. Marker-based gait event detection was used instead of force-based detection due to transient artifacts in the vertical ground reaction force signals produced by rapid belt accelerations, reducing their reliability. We estimated the center of mass of the pelvis in Visual3D with the default model of the pelvis and we used this as a surrogate for the whole-body center of mass (CoM) [69]. CoM velocity was computed as the first derivative of CoM position.

**Fig 3:**
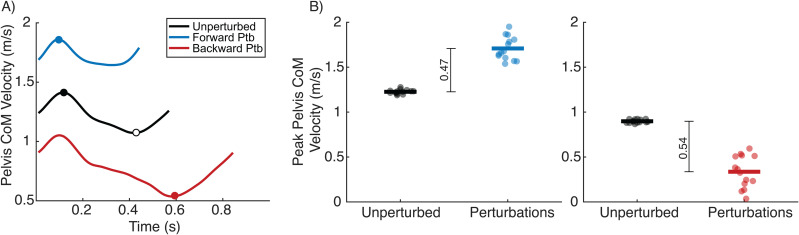
Example of the calculation of CoM velocity responses to perturbations. A) Example of anterior-posterior pelvis CoM velocity relative to the treadmill belt during an unperturbed step (black), a forward perturbation (blue), and a backward perturbation (red). The circles indicate the peak forward and backward CoM velocities. B) Example of peak forward resultant CoM velocities (left panel) during unperturbed and forward perturbation steps, and peak backward CoM velocities (right panel) during unperturbed and backward perturbation steps. Each point represents one step. Horizontal lines represent mean values. The overall CoM response to forward perturbations was computed as the difference between the mean peak forward CoM velocity during forward perturbations and unperturbed walking (black vertical line, left panel). The overall response to backward perturbations was computed as the magnitude of the difference between the mean peak backward CoM velocity during backward perturbations and unperturbed walking (black vertical line, right panel).

Previous work suggests that CoM velocity may be an important sensory signal for perception of treadmill perturbations [39]. Therefore, to test whether the change in CoM velocity during the perturbations may explain inter-individual differences in the preference between the two perturbation directions, we analyzed the peak pelvis CoM velocity during the first forward-backward perturbation pair of each staircase. We chose the first pair because both perturbations were of equal size, in terms of the change in speed from self-selected walking speed, at the beginning of our staircase algorithm. This yielded 14 total perturbations in each direction per day of testing. We first computed the resultant pelvis CoM velocity during each step as the square root of the sum of the squares of the velocities in the anterior-posterior, mediolateral, and vertical directions. Anterior-posterior velocity was computed relative to the treadmill belt. We then identified the peak forward and backward pelvis CoM velocity during the forward and backward perturbations, respectively (Fig 3A). We also computed peak forward and backward pelvis CoM velocities for 14 steps of unperturbed walking from the baseline trial. Next, we characterized the effect of the forward perturbations on the CoM as the magnitude of the difference between the average peak pelvis CoM velocity during all 14 forward perturbations and 14 unperturbed steps (Fig 3B). Similarly, for backward perturbations, we computed the magnitude of the difference between the average peak negative pelvis CoM velocity during backward perturbations and unperturbed steps.

### Statistical analysis

We used t-tests to test for potential differences in walking speed, MiniBESTest, ABC scores, and minutes of exercise per week between the young and older adults. In case of non-normality in the data, we used the Mann-Whitney U-test for independent samples. Normality was assessed using the Shapiro-Wilk test. Effect sizes are reported as rank-biserial correlation for between-group non-parametric comparisons [70,71], Cohen’s *d* for independent-samples comparisons [72], and Cohen’s *dz* for within-subject effects [72,73].

To quantify sensitivity to perturbation direction, we fit a linear mixed effects model with preferred backward perturbation (*Bwd*) magnitude as the dependent variable, forward perturbation (*Fwd*) magnitude as the independent variable, an interaction between *Fwd* magnitude and age group, and random slopes for participants (Equation 1).


$${Bwd}_{ij}=\left(\beta_{\text{1}}+\;\beta_{\text{2}}\times {AgeGroup}_{i}+\;b_{i}\right)\;\times \;{Fwd}_{ij}$$ (1)

Here, *i* refers to the participant number, *j* refers to the staircase number, AgeGroup is 0 for young and 1 for older adults. β_1_ and β_2_ represent the regression coefficients for the *Fwd* magnitude and the interaction term between *Fwd* and age group, respectively. *b*_*i*_ represents the random effect of participants.

The intercept of this model was set to 0, as a non-zero intercept here implies a preference for some backward perturbation magnitude even in the absence of a forward perturbation, which is not a meaningful or plausible interpretation. Because including a main effect of age group would yield a non-zero intercept, only the interaction term between *Fwd* magnitude and age group was included in the model. The term in the parentheses in Equation 1 then represents the slope of the relationship between forward perturbations and subjectively equivalent backward perturbations, which indicates the direction and strength of the preference for a perturbation direction. The final slope for each participant was calculated as a sum of the fixed and random effects. Slopes less than 1 imply a preference for forward perturbations, slopes greater than 1 imply a preference for backward perturbations, and a slope equal to 1 implies indifference between the two directions. Prior to interpreting model results, we assessed key assumptions of linear mixed-effects models. We visually inspected residuals versus fitted values to assess homoscedasticity and plotted quantile-quantile (Q-Q) plots of residuals to check for normality. These diagnostic checks indicated no major violations of modeling assumptions.

To determine the reliability of these slopes as measures of sensitivity to perturbation direction, we fit this model separately to data from a second day of testing. We computed the intra-class correlation coefficient (ICC [1,2]) between the individual slopes obtained on the two days as a measure of the absolute agreement between them. Additionally, to test for the presence of a bias in slopes on Day 2 relative to Day 1, we included testing day as a covariate in the mixed effects model described above (Equation 2). This two-step approach allowed us to first evaluate the relationship between forward and backward perturbations separately on each day, and then assess whether the inclusion of testing day systematically altered this relationship.


$${Bwd}_{ij}=\left(\beta_{\text{1}}+\;\beta_{\text{2}}\times {AgeGroup}_{i}+\;\beta_{\text{3}}\times Day+\;b_{i}\right)\;\times \;{Fwd}_{ij}$$ (2)

The ages of our older adult participants had a wide range, with the lowest being 66 and the oldest being 84 years of age. To account for potential age-dependent differences within this group that may not be captured when grouping them together, we performed an exploratory analysis with the older adults wherein we replaced the age group variable in equation 1 with age as a continuous variable. This allowed us to test if the older adults on the higher end of the age range in this group differed from those on the lower end. We performed this analysis separately with data from the two days of testing.

To investigate the influence of State Anxiety responses to perturbations on people’s preferences for backward versus forward perturbations, we first determined if these responses differed between the two perturbation types and age groups. Because anxiety scores were aggregated Likert scale responses, we used a generalized linear model with a quasi-Poisson error distribution to account for the skew and overdispersion observed in this data. This model included the state anxiety scores as the response variable and perturbation type, age group, and the interaction between them as the predictors. Next, we applied a linear mixed effects model similar to the ones described above in equations 1 and 2 and included the difference in total state anxiety scores between backward and forward perturbations as a covariate (Equation 3). Because state anxiety was only assessed on Day 1, this model only included data from this day.


$${Bwd}_{ij}=\left(\beta_{\text{1}}+\;\beta_{\text{2}}\times {Anxiety\_Diff}_{i}+\;b_{i}\right)\;\times \;{Fwd}_{ij}$$ (3)

Next, we analyzed the pelvis CoM velocity responses to forward and backward perturbations to determine if the difference in these responses between perturbation directions may influence the observed preferences. First, we used a linear model to test for an effect of perturbation type, age group, testing day, and their interactions on changes in peak pelvis CoM velocity relative to baseline. Then, we applied a linear mixed effects model similar to that described above in equation 3, and included the difference between pelvis CoM velocity responses to backward and forward perturbations as a covariate (Equation 4). We applied this model separately to data from the two days of testing. Additionally, we assessed the effect of repeated exposure to perturbations on this difference between responses to backward and forward perturbations using a linear mixed effects model with this difference as the outcome, day of testing as a fixed effect, and a random intercept for participant.


$${Bwd}_{ij}=\left(\beta_{\text{1}}+\;\beta_{\text{2}}\times {vCoM\_Diff}_{i}+\;b_{i}\right)\;\times \;{Fwd}_{ij}$$ (4)

Lastly, to explore whether individual walking speed influenced the bias in perceived risk between forward and backward perturbations, we conducted an exploratory analysis assessing the relationship between participants’ model-derived slopes and their self-selected treadmill walking speeds (SSWS). We used linear models with slope as the outcome, and SSWS, age group and their interaction as predictors. Separate models were applied to slope values derived from day 1 and day 2 of testing.

For the models above, we assessed multicollinearity among fixed effects and found that all variance inflation factors (VIFs) were approximately 1 and correlations among predictors near zero, indicating no collinearity concerns [74]. All statistical analyses were performed using R (version 4.3.1) within RStudio (version 2023.03.0 + 386).

## Results

Our clinical assessments revealed some aspects of physical capacity and activity that were significantly different between our young and older adults, while others were not (Table 1). Our older adults had lower MiniBESTest scores than the young adults, with the observed effect size indicating a large between-group difference (Wilcoxon rank-sum W = 370, p < 0.001; rank-biserial r = 0.85, 95% CI [0.71, 0.92]). The number of participants who experienced a fall in the 12 months preceding the study was also higher among older adults (n = 10) than young adults (n = 7). However, while all falls among the young adults occurred during sports-related activities, 6 out of 10 older adult fallers reported experiencing falls during activities of daily living (e.g., walking on a sidewalk, navigating stairs, walking a dog). Despite the presence of between-group differences in balance and falls, there were no between-group differences in walking speed (p = 0.122), balance confidence scores (p = 0.284), or physical activity (p = 0.100). No participants experienced a fall during the study, and all participants completed both sessions without adverse events or withdrawals.

**Table 1:** Participant characteristics.

	Young Adults (n = 20)	Older Adults (n = 20)	p-value
Age (Years; Mean ± SD)	27 ± 5	71 ± 4	–
Sex	11F/9M	12F/8M	–
Height (m)	1.69 ± 0.09	1.69 ± 0.09	–
Weight (kg)	69.30 ± 12.72	70.78 ± 13.87	–
Treadmill Walking Speed (m/s; Mean ± SD)	1.02 ± 0.13	1.09 ± 0.15	0.122
Number of Fallers	7	10	–
MiniBESTest Total Score (Median (IQR))	28 (IQR 27–28)	26 (IQR 25–26)	< 0.001
ABC Scale Score (Median (IQR))	96 (IQR 90–100)	95 (IQR 87–97)	0.284
Weekly physical activity (minutes; Mean ± SD)	284 ± 216	427 ± 296	0.100

**Fig 4:**
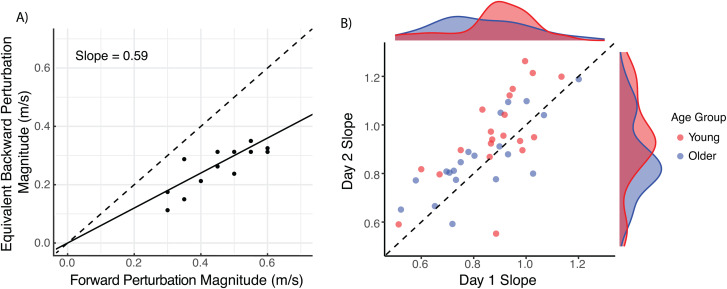
Scatterplots of forward-backward perturbation equivalence points. A) Scatterplot of equivalence points for one representative participant during a single visit. The black dashed line is the unity line, and the black solid line is the linear fit from the mixed effects model. For this participant, all equivalence points lie below the unity line, and the slope is less than one, indicating that forward perturbations were preferred to backward ones. B) Scatterplot of slopes obtained on Day 1 and Day 2 for all participants. Each point represents one participant, with red indicating young and blue indicating older adults. The black dashed line is the unity line. The density plots show the distribution of the slopes on Day 1 (x-axis) and Day 2 (y-axis) for both young (red) and older (blue) adults.

We quantified differences in the sensitivity to perturbation direction by estimating the slopes of a linear model relating forward perturbation magnitude to the subjectively equivalent backward perturbation. Assumptions of homoscedasticity and normality of residuals were verified through visual inspection of residual and Q-Q plots, with no evidence of model misspecification. Slopes less than 1 implied a preference for forward-falling perturbations, slopes greater than 1 implied a preference for backward-falling perturbations, and slopes equal to 1 implied indifference to perturbation direction (Fig 4A). We found a significant association between forward perturbation magnitude and the equally preferred backward perturbation on both days, with slopes generally less than 1, indicating a preference for forward over backward perturbations (Day 1: β = 0.88, SE = 0.04, p < 0.001; Day 2: β = 0.96, SE = 0.04, p < 0.001). There was no significant interaction between forward perturbation size and age group, suggesting that the strength of the sensitivity to perturbation direction was not different between young and older adults (Day 1: p = 0.347; Day 2: p = 0.133). In assessing the variance explained by our model, the marginal R^2^ (representing variance explained by the fixed effects only) on days 1 and 2 was 0.32 and 0.34, respectively. The conditional R^2^ (representing variance explained by both fixed and random effects) on the two days was 0.58 and 0.61, respectively. These results suggest that overall, our sample of young and older adults was sensitive to perturbation direction with a preference for forward over backward perturbations, with no difference in sensitivity between age groups. Participants’ subjective responses indicated that they chose perturbations that felt “easier to recover from” (n = 16), felt “less intense” (n = 7), or felt more comfortable or more likeable (n = 16). Several young and older adults reported perceiving the backward-falling perturbations to be worse and choosing them only when the forward perturbation was much larger than the backward perturbation (n = 16).

We next examined the stability of people’s preferences for forward versus backward perturbations by repeating the staircase procedure on a second day and comparing the model slopes on day 2 to those observed on day 1 (Fig 4B). On day 1, 17 out of 20 young adults and 16 out of 20 older adults had slopes less than 1 (young adults = 0.88 ± 0.15; older adults = 0.83 ± 0.17). On day 2, 13 out of 20 young adults and 15 out of 20 older adults had slopes less than 1 (young adults = 0.96 ± 0.19; older adults = 0.87 ± 0.16). The two-way ICC coefficient was 0.69 (95% confidence interval = 0.45–0.83), implying moderate reliability of model slopes across two days of testing. There was a significant interaction effect of trip magnitude and testing day on preferred slip magnitudes, such that model slopes were greater on day 2 than day 1 (β = 0.06, p < 0.001), with a moderate within-subject effect size (Cohen’s *dz* = 0.44, 95% CI [0.10, 0.77]). These results suggest a reduction in sensitivity to perturbation direction on day 2, with slopes increasing towards 1.

To account for the wide age range among the older adults, we performed an exploratory analysis by treating age as a continuous variable in equation 1. However, on both days of testing, there was no significant interaction effect between trip magnitude and age on the preferred slip magnitude (Day 1: p = 0.31; Day 2: p = 0.97). Therefore, there did not appear to be any differences in the preference between forward and backward balance disturbances between the older adults on the lower and the higher end of the age range in our sample.

**Fig 5:**
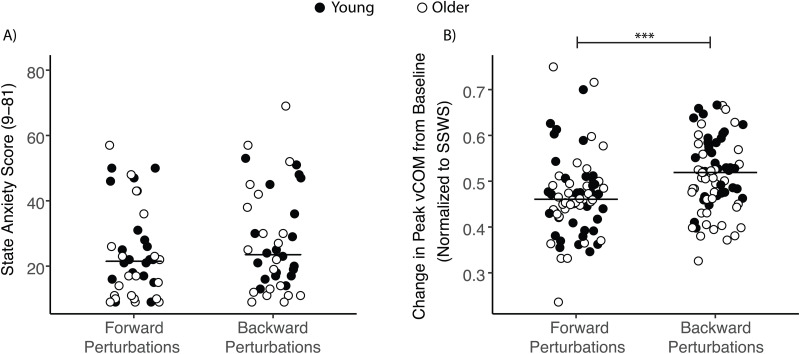
State anxiety and CoM velocity responses to forward and backward perturbations. A) Scatterplot of State Anxiety scores in response to forward and backward perturbations for young (filled circles) and older (open circles) adults. Horizontal lines represent group medians. B) Scatterplot of change in peak CoM velocity from baseline in response to forward and backward perturbations, normalized to self-selected walking speed (SSWS), for young (filled circles) and older (open circles) adults. Change in peak CoM velocity from baseline walking was higher in response to backward perturbations than forward ones (***p < 0.001), with no difference between age groups.

Next, we evaluated if differences in the emotional response to forward-falling and backward-falling perturbations as measured by the State Anxiety Scale might explain people’s preferences. Because this was a 9-item, 9-point Likert scale, the scores were between 9 and 81. There was no effect of perturbation direction (p = 0.774), age group (p = 0.198), or their interaction (p = 0.417) on state anxiety scores (Fig 5A). There was also no interaction effect of forward perturbation magnitude and the difference in state anxiety response between the two perturbation directions on the preferred backward perturbation magnitudes on day 1 (p = 0.345). Thus, people’s preferences were not explained by differences in self-reported anxiety.

Next, we evaluated if differences in the biomechanical effects of backward versus forward treadmill perturbations could explain inter-individual differences in their preferences. Overall, backward perturbations led to greater whole-body disturbances than forward ones, as measured by the changes in pelvis CoM velocity relative to unperturbed walking (Fig 5B). We found a main effect of perturbation direction on peak pelvis CoM velocity relative to baseline, indicating that there was a larger change in peak pelvis CoM velocity in response to backward perturbations than forward ones (β = 0.08, p < 0.001). This main effect of perturbation direction was moderate in magnitude (paired Cohen’s *dz* = 0.54, 95% CI [0.29,0.79]). There was no between-group difference in changes in peak pelvis CoM velocity (p = 0.914), no main effect of testing day (p = 0.715), and no interaction between these variables. There was also no interaction effect of forward perturbation magnitude and the difference between peak pelvis CoM velocity between perturbation directions on the preferred backward perturbation magnitudes on both day 1 (p = 0.860) and day 2 (p = 0.636). Additionally, the difference in the pelvis CoM velocity response to backward and forward perturbations was smaller on day 2 than day 1 (β = −0.03, p < 0.01), with a moderate within-subject effect size (Cohen’s *dz* = 0.50, 95% CI [0.16, 0.84]).

Lastly, we conducted exploratory linear models to investigate the relationship between treadmill walking speeds and participants’ preference for a perturbation direction, as quantified by their model-derived slopes. We performed this analysis separately for slopes obtained on day 1 and day 2 of testing. In both models, walking speed was a significant predictor of slope (Day 1: β = 0.58, p = 0.041; Day 2: β = 0.63, p = 0.038), while the effects of age group and its interaction with walking speeds were not statistically significant. Given the observed range of walking speeds in our participants (0.78 - 1.41m/s) and the range of derived slopes (0.51–1.26), these coefficients suggest that faster walking speeds were associated with slightly higher slope values, indicating a reduced bias towards forward-falling treadmill trips in individuals who walked faster.

## Discussion

When we experience losses of balance while walking, these experiences impact subsequent decisions about how and where we walk. However, little is known about how we process the precise characteristics of experienced losses of balance and if this evaluation process varies in older age when fall-related injuries may have more devastating consequences. As forward-falling trips and backward-falling slips during walking are the most common causes of falls in both young and older adults, we used a two-alternative, forced-choice procedure to determine how people evaluated the relative risk between forward-falling trips and backward-falling slips, delivered using a split-belt treadmill. By measuring points of subjective equality over multiple perturbation sizes, we determined biases in people’s perception of risk associated with these two directions of balance disturbances. Both young and older adults perceived backward perturbations to be riskier than forward ones, but this perceptual bias did not differ between age groups. Our findings provide a first step towards understanding how people evaluate physical risk while walking, which may ultimately inform how prior experiences shape fall-related risk-taking and decision-making.

While previous studies have not directly compared the perception of trips and slips, prior work has demonstrated that treadmill-generated backward-falling slips generate larger whole-body CoM displacements [33,34] and quicker stepping responses [35] compared to forward-falling trips. Backward-falling gait disturbances demand rapid braking or retraction of the slipping foot [28] and coordinated trailing leg support [75], while placing tighter control requirements on the trunk [76,77]. This potentially allows for less flexibility in recovery strategies, as the trunk undergoes rapid extension, generating backward angular momentum that must be quickly arrested to prevent a fall [76,77]. People have also been reported to fall more frequently in response to backward-falling slips versus forward-falling trips of the same magnitude when performing perturbation-based training [12,53,54,78]. These results suggest that backward losses of balance may generally be more difficult to recover from than those in the forward direction. Consistent with these results, we found that the change in peak CoM velocity relative to unperturbed walking was higher during backward than forward treadmill perturbations. In our study, the pelvis CoM was used as a surrogate for whole-body CoM kinematics and may not have fully captured the dynamics of the entire body during balance recovery. A strength of our analysis was that pelvis CoM velocity was only compared between perturbations in the two directions that were equal in terms of change in treadmill belt speed. Our results complement prior observations by establishing that people also perceive backward-falling treadmill slips to be riskier.

Contrary to our hypothesis, the degree of sensitivity to perturbation direction was not different between our young and older adult participants, nor did the changes in CoM velocity in response to perturbations differ between groups. These results are contrary to previous work that has shown that older adults use more ineffective strategies and are less able to successfully recover from both types of perturbations than young adults [14,33,55–58]. Although our older adults had poorer balance than our young adults, as measured by the MiniBESTest, both groups were similar with respect to self-reported physical activity, balance confidence, and walking speed. 84% of our older adults reported engaging in at least 150 minutes of exercise per week, which is the recommended amount of exercise according to the 2008 Physical Activity Guidelines for Americans [79]. This percentage of older adults engaged in exercise is much higher than that reported for the general population of older adults (27.3% − 44.3%) [80]. These findings suggest that our sample may not be representative of the general older adult population. While some older adults in our sample reported experiencing a fall in the previous year, this alone did not differentiate them from non-fallers in terms of sensitivity to perturbation direction, CoM velocity responses to perturbations and state anxiety. Future work should examine if older adults with lower balance confidence and physical activity or fitness levels exhibit greater sensitivity to perturbation direction, and if so, if this is then related to their self-reported fear of falling or fall history [81–83]. Interestingly, in an exploratory analysis, we found that individuals who walked faster tended to show a slightly reduced bias towards forward perturbations, suggesting that perceived risk may be influenced by walking speed or ability. However, this relationship was modest and consistent across age groups, warranting further investigation in larger, more diverse samples. Lastly, our treadmill perturbations were controlled in magnitude and not sufficiently large to induce frequent falls. It is possible that the use of larger forward and backward perturbations may have revealed an effect of age, if present, such that differences in sensitivity to perturbation direction only emerge in near-fall or actual fall situations.

We found that the sensitivity to perturbation direction was stable across two days of testing such that those who found backward-falling treadmill slips to be riskier on the first day also found them to be riskier on the second day. This was supported by a moderate level of reliability (ICC = 0.69), indicating that while individual differences in sensitivity were relatively consistent, there was also meaningful variability between days. Specifically, the degree of sensitivity was lower on the second day. Previous work has shown that both young and older adults are able to improve their reactive responses to these perturbations with training [55,84,85]. It is unclear whether this adaptation of reactive responses is associated with a reduction in perceived risk or balance threat posed by these losses of balance. Although our study was not designed to explicitly test for learning effects, we found that the difference in participants’ CoM velocity-based responses between backward and forward perturbations reduced from the first to the second day of testing. This suggests a potential adaptation of biomechanical responses across days, possibly reflecting a decreased destabilizing effect of backward perturbations with repeated exposure. Emotional arousal and anxiety have been shown to decrease with repeated exposure to the threat of standing perturbations [86]. Therefore, it is possible that our observation of reduced sensitivity to perturbation direction on the second day of testing may be due to a reduction in the perceived risk of treadmill slips due to repeated exposure on the first day. Future studies should assess both emotional and physical responses at various time points during protocols that require experiencing multiple gait perturbations to better understand the underlying mechanisms of any observed behavioral responses to the perturbations. Additionally, our observed level of reliability suggests that our measure may not yet be suitable as a stand-alone clinical assessment tool, as it may not provide a fully stable estimate of an individual’s perceived risk bias in a single session. Averaging performance across multiple sessions or incorporating repeated assessments may provide a more reliable estimate for clinical applications.

Several potential factors may lead to differences in people’s perceived risk associated with forward-falling versus backward-falling gait perturbations. First, if the two directions of perturbations elicit distinct emotional responses, these responses may inform an individual’s risk perception and choices. In behavioral economics, this is referred to as affect-based decision-making, where affect is the overall feeling of “good” or “bad” associated with a stimulus [8]. Anxiety in response to postural perturbations has been found to be correlated with self-reported perceptions of postural instability in both young [45,87] and older adults [87]. Therefore, we hypothesized that measures of state anxiety in response to backward-falling treadmill slips and forward-falling treadmill trips would correlate with people’s preferences between the two. However, we found no differences in self-reported state anxiety between the two perturbation types and between the two age groups. We also did not find a correlation between state anxiety and preference between the perturbation directions. This may be in part due to the limitations of self-reports themselves [88], as participants may use different frames of reference for their responses. Therefore, some participants may have reported their anxiety in the context of falling such that if they did not fall, they reported low anxiety. Since our perturbations did not cause falls, this could have resulted in similar anxiety scores between forward-falling and backward-falling perturbations. This limitation can be addressed by approximating anxiety using an objective and instantaneous measure of arousal, such as electrodermal activity [89–91], which has previously been found to be sensitive to postural threat [46]. In addition, while our study included the Activities-specific Balance Confidence scale as a measure of perceived balance ability, future work could incorporate additional psychological assessments, such as fear of falling and trait anxiety measures. When combined with measures of state anxiety, these may provide a richer context for understanding an individual’s risk perception process.

A second potential factor that may explain people’s preference between treadmill trips and slips is the actual physical disturbance of the body to the two perturbation directions. A study of perception thresholds of treadmill slips found that CoM velocity may be an important source of sensory information used by the nervous system to detect slips [39]. Therefore, we hypothesized that peak CoM velocity during forward and backward treadmill perturbations may be used as a source of sensory information that informs perceptions of severity and, thus, may explain people’s preferences. However, we found that relative changes in peak CoM velocity from baseline during these two types of perturbations did not correlate with our measure of preference. It is possible that a combination of proprioceptive signals related to foot, lower limb, and head kinematics may better explain people’s preferences. A final consideration is that in our experimental setup, handlebars were available towards the front of the treadmill. While participants were verbally discouraged from constant use of this external support, some did still use them in anticipation of perturbations. Therefore, the measured CoM velocity following perturbations was occasionally confounded by the use of support bars. Future studies can either remove support bars, if possible, or record their use so that they can be accounted for.

While our two-alternative forced-choice question was designed to evoke naturalistic judgements of perceived risk or severity (“If you had to repeat one of these perturbations, which one would you choose?”), we recognize that this phrasing may not have directly captured perceived fall risk for all participants. Although most participants reported making their decisions based on perceived difficulty or safety of the perturbation, some responses may have been influenced by alternative motivations that were not reported. This is a potential source of variability in the decision-making process used by our participants. Future studies may directly compare preference-based responses with explicit risk ratings and incorporate verbal probes after each choice to better understand participants’ reasoning in real time. Finally, while our sample size was consistent with prior studies of perception of locomotor perturbations [38,39], no formal sample size calculation was performed. Therefore, the study may have been underpowered to detect smaller effects, and replication in larger, more diverse cohorts will be important to confirm and extend these findings.

## Conclusion

Both young and older adults perceive slips simulated using a split-belt treadmill to be riskier than trips, although the underlying reasons for this preference remain to be understood. Measuring the degree of preference for different perturbation types, along with a person’s ability to recover from them, may help identify activities that are most likely to cause falls. This information could be used to personalize training programs in accordance with an individual’s risk perception and balance capacity. Subsequent studies should also examine how the characteristics of experienced losses of balance inform subsequent behavioral decisions. For example, epidemiological data on the prevalence of falls is often collapsed across the causes or perceived causes of falls such that slips and trips are grouped together [9,11]. In future studies, it may be valuable to consider the type of loss of balance when characterizing falls and then determine if this information predicts subsequent changes in behavior.

## References

[1] Hawkins K, Musich S, Ozminkowski RJ, Bai M, Migliori RJ, Yeh CS. The burden of falling on the quality of life of adults with Medicare supplement insurance. J Gerontol Nurs. 2011;37(8):36–47. doi: 10.3928/00989134-20110329-03 21485987

[2] Jiang H, Yuan H, Tee S, Lam Nogueira OCB. Perspectives and experiences of community-dwelling older adults who experience falling: A qualitative meta-synthesis. Int J Nurs Sci. 2024. 10.1016/j.ijnss.2024.03.009 PMC11064561 38707695

[3] Burns ER, Stevens JA, Lee R. The direct costs of fatal and non-fatal falls among older adults — United States. J Safety Res. 2016;58:99–103. 27620939 10.1016/j.jsr.2016.05.001 PMC6823838

[4] Jain S, Schweighofer N, Finley JM. Aberrant decision-making as a risk factor for falls in aging. Front Aging Neurosci. 2024;16:1384242. doi: 10.3389/fnagi.2024.1384242 38979111 PMC11229407

[5] Feldman F, Chaudhury H. Falls and the physical environment: a review and a new multifactorial falls-risk conceptual framework. Can J Occup Ther. 2008;75(2):82–95. 18510252 10.1177/000841740807500204

[6] Kuljeerung O, Lach HW. Extrinsic and Behavioral Fall Risk Factors in People With Parkinson’s Disease: An Integrative Review. Rehabil Nurs. 2021;46(1):3–10. doi: 10.1097/rnj.0000000000000265 33395012

[7] Tobler PN, Weber EU. Valuation for risky and uncertain choices. Neuroeconomics. Elsevier. 2014. p. 149–72.

[8] Slovic P, Finucane ML, Peters E, MacGregor DG. Risk as analysis and risk as feelings: some thoughts about affect, reason, risk, and rationality. Risk Anal. 2004;24(2):311–22. doi: 10.1111/j.0272-4332.2004.00433.x 15078302

[9] Heijnen MJH, Rietdyk S. Falls in young adults: perceived causes and environmental factors assessed with a daily online survey. Hum Mov Sci. 2016;46:86–95. 26741254 10.1016/j.humov.2015.12.007

[10] Li W, Keegan THM, Sternfeld B, Sidney S, Quesenberry CP Jr, Kelsey JL. Outdoor falls among middle-aged and older adults: a neglected public health problem. Am J Public Health. 2006;96(7):1192–200. doi: 10.2105/AJPH.2005.083055 16735616 PMC1483851

[11] Talbot LA, Musiol RJ, Witham EK, Metter EJ. Falls in young, middle-aged and older community dwelling adults: perceived cause, environmental factors and injury. BMC Public Health. 2005;5:86. doi: 10.1186/1471-2458-5-86 16109159 PMC1208908

[12] Okubo Y, Brodie MA, Sturnieks DL, Hicks C, Carter H, Toson B, et al. Exposure to trips and slips with increasing unpredictability while walking can improve balance recovery responses with minimum predictive gait alterations. PLoS One. 2018;13(9):e0202913. doi: 10.1371/journal.pone.0202913 30226887 PMC6143193

[13] Grabiner MD, Koh TJ, Lundin TM, Jahnigen DW. Kinematics of Recovery From a Stumble. J Gerontol. 1993;48(3):M97-102. 10.1093/geronj/48.3.m97 8482818

[14] Senden R, Savelberg HHCM, Adam J, Grimm B, Heyligers IC, Meijer K. The influence of age, muscle strength and speed of information processing on recovery responses to external perturbations in gait. Gait Posture. 2014;39(1):513–7. doi: 10.1016/j.gaitpost.2013.08.033 24119777

[15] Bhatt T, Wening JD, Pai Y-C. Influence of gait speed on stability: recovery from anterior slips and compensatory stepping. Gait Posture. 2005;21(2):146–56. doi: 10.1016/j.gaitpost.2004.01.008 15639393

[16] Bruijn SM, Sloot LH, Kingma I, Pijnappels M. Contribution of arm movements to balance recovery after tripping in older adults. J Biomech. 2022;133:110981. 35123206 10.1016/j.jbiomech.2022.110981

[17] de Boer T, Wisse M, van der Helm FCT. Mechanical analysis of the preferred strategy selection in human stumble recovery. J Biomech Eng. 2010;132(7):071012. doi: 10.1115/1.4001281 20590290

[18] Eng JJ, Winter DA, Patla AE. Strategies for recovery from a trip in early and late swing during human walking. Exp Brain Res. 1994;102(2):339–49. doi: 10.1007/BF00227520 7705511

[19] Hwang S, Tae K, Sohn R, Kim J, Son J, Kim Y. The balance recovery mechanisms against unexpected forward perturbation. Ann Biomed Eng. 2009;37(8):1629–37. 19472056 10.1007/s10439-009-9717-y

[20] Liu J, Lockhart TE. Age-related joint moment characteristics during normal gait and successful reactive-recovery from unexpected slip perturbations. Gait Posture. 2009;30(3):276–81. doi: 10.1016/j.gaitpost.2009.04.005 19581088 PMC3716287

[21] Pijnappels M, Reeves ND, Maganaris CN, van Dieën JH. Tripping without falling; lower limb strength, a limitation for balance recovery and a target for training in the elderly. J Electromyogr Kinesiol. 2008;18(2):188–96. doi: 10.1016/j.jelekin.2007.06.004 17761436

[22] Pijnappels M, Bobbert MF, van Dieën JH. Contribution of the support limb in control of angular momentum after tripping. J Biomech. 2004;37(12):1811–8. 15519588 10.1016/j.jbiomech.2004.02.038

[23] Pijnappels M, Bobbert MF, van Dieën JH. Control of support limb muscles in recovery after tripping in young and older subjects. Exp Brain Res. 2005;160(3):326–33. doi: 10.1007/s00221-004-2014-y 15322782

[24] Pijnappels M, Bobbert MF, van Dieën JH. How early reactions in the support limb contribute to balance recovery after tripping. J Biomech. 2005;38(3):627–34. doi: 10.1016/j.jbiomech.2004.03.029 15652564

[25] Tokur D, Grimmer M, Seyfarth A. Review of balance recovery in response to external perturbations during daily activities. Hum Mov Sci. 2020;69:102546. 31989948 10.1016/j.humov.2019.102546

[26] Roos PE, McGuigan MP, Trewartha G. The role of strategy selection, limb force capacity and limb positioning in successful trip recovery. Clin Biomech (Bristol). 2010;25(9):873–8. doi: 10.1016/j.clinbiomech.2010.06.016 20667634

[27] Schillings AM, van Wezel BMH, Mulder TH, Duysens J. Muscular responses and movement strategies during stumbling over obstacles. J Neurophysiol. 2000;83(4):2093–102. 10758119 10.1152/jn.2000.83.4.2093

[28] Redfern MS, Cham R, Gielo-Perczak K, Grönqvist R, Hirvonen M, Lanshammar H. Biomechanics of slips. Ergonomics. 2001;44(13):1138–66. 11794762 10.1080/00140130110085547

[29] Cham R, Redfern MS. Lower extremity corrective reactions to slip events. J Biomech. 2001;34(11):1439–45. 11672718 10.1016/s0021-9290(01)00116-6

[30] Jayadas A, Smith J. Identification of effective and ineffective reactive movements when attempting to recover from a slippery perturbation. 2022.

[31] Moyer BE, Redfern MS, Cham R. Biomechanics of trailing leg response to slipping - evidence of interlimb and intralimb coordination. Gait Posture. 2009;29(4):565–70. doi: 10.1016/j.gaitpost.2008.12.012 19196513 PMC4878699

[32] Marigold DS, Bethune AJ, Patla AE. Role of the unperturbed limb and arms in the reactive recovery response to an unexpected slip during locomotion. J Neurophysiol. 2003;89(4):1727–37. doi: 10.1152/jn.00683.2002 12611998

[33] Lockhart TE, Woldstad JC, Smith JL. Effects of age-related gait changes on the biomechanics of slips and falls. Ergonomics. 2003;46(12):1136–60. doi: 10.1080/0014013031000139491 12933077 PMC2891178

[34] Bhatt T, Pai YC. Generalization of gait adaptation for fall prevention: from moveable platform to slippery floor. J Neurophysiol. 2009;101(2):948–57. doi: 10.1152/jn.91004.2008 19073804 PMC2657073

[35] Lee B-C, Kim C-S, Seo K-H. The Body’s Compensatory Responses to Unpredictable Trip and Slip Perturbations Induced by a Programmable Split-Belt Treadmill. IEEE Trans Neural Syst Rehabil Eng. 2019;27(7):1389–96. doi: 10.1109/TNSRE.2019.2921710 31180863

[36] Lee B-C, Martin BJ, Thrasher TA, Layne CS. The Effect of Vibrotactile Cuing on Recovery Strategies From a Treadmill-Induced Trip. IEEE Trans Neural Syst Rehabil Eng. 2017;25(3):235–43. doi: 10.1109/TNSRE.2016.2556690 28333619

[37] Golyski PR, Vazquez E, Leestma JK, Sawicki GS. Onset timing of treadmill belt perturbations influences stability during walking. J Biomech. 2022;130:110800. 34864443 10.1016/j.jbiomech.2021.110800

[38] Liss DJ, Carey HD, Yakovenko S, Allen JL. Young adults perceive small disturbances to their walking balance even when distracted. Gait Posture. 2022;91:198–204. doi: 10.1016/j.gaitpost.2021.10.019 34740056 PMC8671331

[39] Liss DJ, Carey HD, Allen JL. Young adults use whole-body feedback and ankle proprioception to perceive small locomotor disturbances. Hum Mov Sci. 2023;89:103084. doi: 10.1016/j.humov.2023.103084 36989968

[40] Phu S, Sturnieks DL, Song PYH, Lord SR, Okubo Y. Treadmill induced belt-accelerations may not accurately evoke the muscle responses to obstacle trips in older people. J Electromyogr Kinesiol. 2024;75:102857. doi: 10.1016/j.jelekin.2024.102857 38330509

[41] Siragy T, Russo Y, Young W, Lamb SE. Comparison of over-ground and treadmill perturbations for simulation of real-world slips and trips: A systematic review. Gait Posture. 2023;100:201–9. doi: 10.1016/j.gaitpost.2022.12.015 36603326

[42] Siegrist M, Árvai J. Risk perception: reflections on 40 years of research. Risk Anal. 2020;40(S1):2191–206. 32949022 10.1111/risa.13599

[43] Adkin AL, Carpenter MG. New Insights on Emotional Contributions to Human Postural Control. Front Neurol. 2018;9:789. doi: 10.3389/fneur.2018.00789 30298048 PMC6160553

[44] Carpenter MG, Adkin AL, Brawley LR, Frank JS. Postural, physiological and psychological reactions to challenging balance: does age make a difference?. Age Ageing. 2006;35(3):298–303. 16638771 10.1093/ageing/afl002

[45] Cleworth TW, Carpenter MG. Neuroscience Letters. 2016;620:127–31. 27016388 10.1016/j.neulet.2016.03.032

[46] Cleworth TW, Inglis JT, Carpenter MG. Postural threat influences the conscious perception of body position during voluntary leaning. Gait Posture. 2018;66:21–5. doi: 10.1016/j.gaitpost.2018.08.003 30138743

[47] Attwood AS, Ludwig CJH, Penton-Voak IS, Poh J, Kwong ASF, Munafò MR. Effects of state anxiety on gait: a 7.5% carbon dioxide challenge study. Psychol Res. 2021;85(6):2444–52. doi: 10.1007/s00426-020-01393-2 32737585 PMC8357656

[48] Norouzian P, Horslen BC, Martens KAE. The effects of trait and state anxiety on gait in healthy young adults. Exp Brain Res. 2024;242(4):819–28. doi: 10.1007/s00221-024-06800-3 38456925

[49] Bonem EM, Ellsworth PC, Gonzalez R. Age Differences in Risk: Perceptions, Intentions and Domains. J Behav Decis Mak. 2015;28(4):317–30.

[50] Waltrip LH, Joyce JL, Chapman S, Cosentino S, Sunderaraman P. Risk-taking behavior in older adults is related to cognitive outcomes in a domain-specific manner. Alzheimers Dement. 2023;19(S18):e078930.

[51] Rolison JJ, Hanoch Y, Wood S, Liu PJ. Risk-Taking Differences Across the Adult Life Span: A Question of Age and Domain. J Gerontol Ser B. 2014;69(6):870–80. 10.1093/geronb/gbt081 24149517

[52] Roalf DR, Mitchell SH, Harbaugh WT, Janowsky JS. Risk, reward, and economic decision making in aging. J Gerontol Ser B. 2012;67B(3):289–98. 10.1093/geronb/gbr099 PMC3325085 21926401

[53] Crenshaw JR, Bernhardt KA, Achenbach SJ, Atkinson EJ, Khosla S, Kaufman KR, et al. The circumstances, orientations, and impact locations of falls in community-dwelling older women. Arch Gerontol Geriatr. 2017;73:240–7. doi: 10.1016/j.archger.2017.07.011 28863352 PMC5858880

[54] Pitchai P, Dedhia HB, Bhandari N, Krishnan D, D’Souza NRJ, Bellara JM. Prevalence, risk factors, circumstances for falls and level of functional independence among geriatric population - A descriptive study. Indian J Public Health. 2019;63(1):21–6. doi: 10.4103/ijph.IJPH_332_17 30880733

[55] Dijkstra BW, Horak FB, Kamsma YPT, Peterson DS. Older adults can improve compensatory stepping with repeated postural perturbations. Front Aging Neurosci. 2015;7:201. doi: 10.3389/fnagi.2015.00201 26539111 PMC4612504

[56] Liu J. Aging effect on joint moment generation strategy in successful reactive-recovery from unexpected slips. In: Proceedings of the Human Factors and Ergonomics Society Annual Meeting. 2005;1302–5.

[57] Yoo D, An J, Seo K-H, Lee B-C. Aging Affects Lower Limb Joint Moments and Muscle Responses to a Split-Belt Treadmill Perturbation. Front Sports Act Living. 2021;3:683039. doi: 10.3389/fspor.2021.683039 34350396 PMC8326400

[58] Lauretani F, Maggio M, Ticinesi A, Tana C, Prati B, Gionti L, et al. Muscle weakness, cognitive impairment and their interaction on altered balance in elderly outpatients: results from the TRIP observational study. Clin Interv Aging. 2018;13:1437–43. 30174417 10.2147/CIA.S165085 PMC6109650

[59] Franchignoni F, Horak F, Godi M, Nardone A, Giordano A. Using psychometric techniques to improve the Balance Evaluation Systems Test: the mini-BESTest. J Rehabil Med. 2010;42(4):323–31. doi: 10.2340/16501977-0537 20461334 PMC3228839

[60] Powell LE, Myers AM. The Activities-specific Balance Confidence (ABC) Scale. J Gerontol A Biol Sci Med Sci. 1995;50A(1):M28-34. doi: 10.1093/gerona/50a.1.m28 7814786

[61] Liu C, Park S, Finley J. The choice of reference point for computing sagittal plane angular momentum affects inferences about dynamic balance. PeerJ. 2022;10:e13371. doi: 10.7717/peerj.13371 PMC9107787 35582618

[62] Buurke TJW, Liu C, Park S, den Otter R, Finley JM. Maintaining sagittal plane balance compromises frontal plane balance during reactive stepping in people post-stroke. Clin Biomech. 2020;80:105135. 10.1016/j.clinbiomech.2020.105135 PMC8128665 32818902

[63] Hauck LJ, Carpenter MG, Frank JS. Task-specific measures of balance efficacy, anxiety, and stability and their relationship to clinical balance performance. Gait Posture. 2008;27(4):676–82. doi: 10.1016/j.gaitpost.2007.09.002 17942311

[64] CORNSWEET TN. The staircrase-method in psychophysics. Am J Psychol. 1962;75:485–91. doi: 10.2307/1419876 13881416

[65] Wang P, Reynaud A. The Random Step Method for Measuring the Point of Subjective Equality. Vision (Basel). 2023;7(4):74. doi: 10.3390/vision7040074 37987294 PMC10661322

[66] Liu C, Finley JM. Asymmetric gait patterns alter the reactive control of intersegmental coordination patterns in the sagittal plane during walking. PLOS ONE. 2020 May 21;15(5):e0224187. 10.1371/journal.pone.0224187 PMC7241778 32437458

[67] Winter DA. Biomechanics and Motor Control of Human Movement. John Wiley & Sons. 2009.

[68] Reisman DS, Wityk R, Silver K, Bastian AJ. Split-belt treadmill adaptation transfers to overground walking in persons poststroke. Neurorehabil Neural Repair. 2009;23(7):735–44. doi: 10.1177/1545968309332880 19307434 PMC2811047

[69] Havens KL, Mukherjee T, Finley JM. Analysis of biases in dynamic margins of stability introduced by the use of simplified center of mass estimates during walking and turning. Gait Posture. 2018;59:162–7. doi: 10.1016/j.gaitpost.2017.10.002 29031999 PMC5690861

[70] Willson VL. Critical Values of the Rank-Biserial Correlation Coefficient. Educational and Psychological Measurement. 1976;36(2):297–300. doi: 10.1177/001316447603600207

[71] Kerby DS. The Simple Difference Formula: An Approach to Teaching Nonparametric Correlation. Comprehensive Psychol. 2014;3:11.IT.3.1. doi: 10.2466/11.it.3.1

[72] Cohen J. Statistical power analysis for the behavioral sciences. 2nd ed. New York: Routledge. 2013.

[73] Lakens D. Calculating and reporting effect sizes to facilitate cumulative science: a practical primer for t-tests and ANOVAs. Front Psychol. 2013;4. 10.3389/fpsyg.2013.00863 PMC3840331 24324449

[74] Zuur AF. Mixed effects models and extensions in ecology with R. New York, NY: Springer. 2009.

[75] Moyer BE, Redfern MS, Cham R. Biomechanics of trailing leg response to slipping - evidence of interlimb and intralimb coordination. Gait Posture. 2009;29(4):565–70. doi: 10.1016/j.gaitpost.2008.12.012 19196513 PMC4878699

[76] Liu J, Lockhart TE. Trunk angular kinematics during slip-induced falls and activities of daily living - towards developing a fall detector. Proc Hum Factors Ergon Soc Annu Meet. 2009;53(14):892–6.

[77] Liu J, Lockhart TE. Trunk angular kinematics during slip-induced backward falls and activities of daily living. J Biomech Eng. 2014;136(10):101005. doi: 10.1115/1.4028033 25033029 PMC4127473

[78] Okubo Y, Brodie MA, Sturnieks DL, Hicks C, Lord SR. A pilot study of reactive balance training using trips and slips with increasing unpredictability in young and older adults: biomechanical mechanisms, falls and clinical feasibility. Clin Biomech. 2019;67:171–9. 10.1016/j.clinbiomech.2019.05.016 31153101

[79] Physical Activity Guidelines Advisory Committee Report, 2008 to the Secretary of Health and Human Services. PsycEXTRA Dataset. American Psychological Association (APA). 2008. doi: 10.1037/e525442010-001 19178654

[80] Keadle SK, McKinnon R, Graubard BI, Troiano RP. Prevalence and trends in physical activity among older adults in the United States: A comparison across three national surveys. Prev Med. 2016;89:37–43. 27196146 10.1016/j.ypmed.2016.05.009 PMC4969157

[81] Donoghue OA, Cronin H, Savva GM, O’Regan C, Kenny RA. Effects of fear of falling and activity restriction on normal and dual task walking in community dwelling older adults. Gait Posture. 2013;38(1):120–4. doi: 10.1016/j.gaitpost.2012.10.023 23200462

[82] Bruce DG, Devine A, Prince RL. Recreational physical activity levels in healthy older women: the importance of fear of falling. J Am Geriatr Soc. 2002;50(1):84–9. doi: 10.1046/j.1532-5415.2002.50012.x 12028251

[83] Sales M, Levinger P, Polman R. Relationships between self perceptions and physical activity behaviour, fear of falling, and physical function among older adults. Eur Rev Aging Phys Act. 2017;14(1):17. 28943974 10.1186/s11556-017-0185-3 PMC5607601

[84] Allin LJ, Brolinson PG, Beach BM, Kim S, Nussbaum MA, Roberto KA, et al. Perturbation-based balance training targeting both slip- and trip-induced falls among older adults: a randomized controlled trial. BMC Geriatr. 2020;20(1):205. doi: 10.1186/s12877-020-01605-9 32532221 PMC7291462

[85] Chien J-E, Hsu W-L. Effects of dynamic perturbation-based training on balance control of community-dwelling older adults. Sci Rep. 2018;8(1):17231. doi: 10.1038/s41598-018-35644-5 30467355 PMC6250668

[86] Johnson KJ, Zaback M, Tokuno CD, Carpenter MG, Adkin AL. Repeated exposure to the threat of perturbation induces emotional, cognitive, and postural adaptations in young and older adults. Exp Gerontol. 2019;122:109–15. doi: 10.1016/j.exger.2019.04.015 31028840

[87] Castro P, Kaski D, Schieppati M, Furman M, Arshad Q, Bronstein A. Subjective stability perception is related to postural anxiety in older subjects. Gait Posture. 2019;68:538–44. doi: 10.1016/j.gaitpost.2018.12.043 30634135

[88] Junghaenel DU, Broderick JE, Schneider S, May M, Bolton A, McCarrier KP, et al. Frames of reference in self-reports of health, well-being, fatigue, and pain: a qualitative examination. Appl Res Qual Life. 2018;13(3):585–601. doi: 10.1007/s11482-017-9546-3 30344794 PMC6191058

[89] Naveteur J, Freixa I, Baque E. Individual differences in electrodermal activity as a function of subjects’ anxiety. Personal Individ Differ. 1987;8(5):615–26.

[90] Ödegaard Ö. The psychogalvanic reactivity in affective disorders. Br J Med Psychol. 1932;12(2):132–50.

[91] Rahma ON, Putra AP, Rahmatillah A, Putri YSKA, Fajriaty ND, Ain K, et al. Electrodermal activity for measuring cognitive and emotional stress level. J Med Signals Sens. 2022;12(2):155–62. doi: 10.4103/jmss.JMSS_78_20 35755979 PMC9215837

